# Spatial Distribution, Abundance, and Threats to the Indian Pangolin ( *Manis crassicaudata* ) in Buner District, Lesser Himalayas

**DOI:** 10.1002/ece3.73344

**Published:** 2026-05-04

**Authors:** Muhammad Saad, Shahrul Anuar Mohd Sah

**Affiliations:** ^1^ School of Biological Sciences University of Sains Malaysia Penang Malaysia

**Keywords:** abundance, burrow ecology, conservation, elevation gradient, *Manis crassicaudata*, spatial distribution, threats

## Abstract

The Indian pangolin ( *Manis crassicaudata* ), an elusive and endangered mammal, remains poorly studied in Pakistan, particularly in the mountain landscapes of the Lesser Himalayas. This study provides the first detailed ecological baseline of the species' spatial distribution, burrow characteristics, habitat associations, and anthropogenic threats in District Buner, Khyber Pakhtunkhwa, based on systematic field surveys conducted from October 2022 to September 2023. Using a stratified random sampling design, systematic surveys were conducted across 54 km^2^ of surveyed habitat along line transects and associated circular plots, resulting in the documentation of 127 pangolin burrows across four tehsils. These comprised feeding burrows (63.8%), inactive burrows (26.0%), and active resting (living) burrows (10.2%). The burrow‐based abundance patterns indicated that active burrows were primarily recorded between 300 and 700 m elevation, with occasional occurrences up to 1300 m, and were absent above this range. Burrow activity was highest in Mandanr Tehsil and was associated with north‐ and east‐facing slopes, loamy soils, rocky substrates, moderate canopy cover (41%–70%), and dense understory vegetation. Phytohabitat associations showed that resting burrows were frequently linked to *Lantana camara* and *Punica granatum* , whereas feeding burrows were commonly associated with *Dalbergia sissoo* , *Acacia modesta*, and prey‐rich substrates. Burrow concentrations were greatest within ecotonal zones between scrub forest and subtropical pine forest, while areas dominated by *Pinus roxburghii*, *Juglans regia* , and *Quercus incana* showed no evidence of pangolin presence. Two direct sightings and physical remains provided independent confirmation of species presence and suggested localized decline at some sites. Major threats identified through field observations and community surveys included poaching, illegal trade, habitat degradation, marble mining, and unregulated infrastructure development, exacerbated by weak law enforcement and low local awareness. This study provides a robust ecological baseline for *M. crassicaudata* in the Lesser Himalayas of Pakistan and informs targeted, context‐specific conservation interventions.

## Graphical abstract

First quantitative study of the Indian pangolin population in Buner District. GIS mapping revealed uneven spatial distribution across elevation zones.

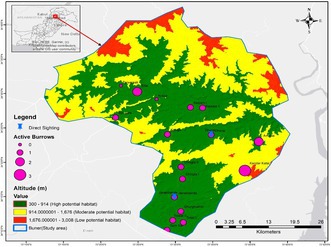

## 1 Introduction

Pangolins are among the most evolutionarily distinct and heavily exploited mammals worldwide. The term “pangolin” is derived from the Malayan word *pen gulling*, meaning “rolling into a ball,” which reflects their characteristic defensive behavior (Pearsall 2002). Commonly known as scaly anteaters, pangolins possess keratinized scales and exhibit a highly specialized myrmecophagous diet consisting primarily of ants and termites (Perera et al. 2017). Globally, nine species are recognized, including four African species (*Phataginus tricuspis*, *P. tetradactyla* , *Smutsia temminckii*, and *S. gigantea* ) and five Asian species ( *Manis crassicaudata* , *M. pentadactyla* , *M. javanica* , *M. culionensis* , and *M. palawanensis*) (Gaubert et al. 2018). All pangolin species are listed under Appendix S1 of the Convention on International Trade in Endangered Species of Wild Fauna and Flora (CITES), prohibiting international commercial trade (CITES 2019). Pangolins are nocturnal, solitary, fossorial, and nonaggressive mammals belonging to the Order Pholidota and Family Manidae (Gray 1821). They are classified as Evolutionarily Distinct and Globally Endangered (EDGE) taxa due to their unique evolutionary history and high extinction risk (Gaubert and Antunes 2005; Lamichhane and Pokhrel 2019; Yasmeen et al. 2021).

The Indian pangolin ( *Manis crassicaudata* ) is distributed across South Asia, ranging from northern and southeastern Pakistan through the Indian subcontinent south of the Himalayas, and extending into Nepal, Bangladesh, Sri Lanka, and northeastern India (Mohapatra et al. 2015). Within Pakistan, the species has been reported from localized and discontinuous populations in Sindh, Balochistan, Punjab, Khyber Pakhtunkhwa, and parts of Azad Jammu and Kashmir (Roberts 1997; Akrim et al. 2017; Mahmood et al. 2019). Although its wide geographic range, its occurrence is highly patchy and spatially variable. The Indian pangolin is currently listed as *Endangered* on the IUCN Red List of Threatened Species (Mahmood et al. 2019) and is afforded the highest level of legal protection through its inclusion in Appendix S1 of CITES, which prohibits international commercial trade. At the national level, it is designated as a Schedule I species under Pakistan's Wildlife (Protection) Amendment Act, 2022, reflecting its elevated vulnerability to poaching and illegal trade.

The Indian pangolin ( *Manis crassicaudata* ) occupies a wide range of habitats, including forests, open plains, hilly terrain, and agricultural landscapes near human settlements (Roberts 1997; Zoological Survey of India 2002). The species typically occurs at low population densities and shows a preference for forested and seminatural habitats (Gaudin et al. 2006). Across its range, it has been recorded in diverse vegetation types, including dry and moist deciduous forests, semievergreen forests, grasslands, and thorn scrub, as well as in abandoned or degraded lands adjacent to villages (Perera et al. 2017). Despite this broad habitat use, pangolin presence is strongly mediated by prey availability, particularly ants and termites, and reduced hunting pressure, indicating ecological flexibility within defined disturbance controls.

The Indian pangolin ( *Manis crassicaudata* ) constructs two functionally distinct burrow types, shallow feeding burrows excavated to access prey and deeper resting burrows used for daytime refuge and breeding (Mahmood et al. 2012). These burrow types can be reliably distinguished by entrance size, depth, and the presence of prey remains. As a fossorial and nocturnal species, the Indian pangolin remains underground during daylight hours and emerges at night to forage. Within its range, it occupies a variety of environments, including barren hills, subtropical thorn forests, grasslands, and seminatural landscapes, provided that suitable soils, shelter features (e.g., rock formations and water sources), and abundant ant and termite prey are available (Gaudin et al. 2006; Perera et al. 2022; Gu et al. 2023).

Though this ecological flexibility, the species is increasingly threatened by habitat degradation, human–pangolin conflict, and intensive poaching driven by demand for meat and scales used in traditional medicine (Mahmood et al. 2012; Challender et al. 2015; Irshad et al. 2015). Pangolins are particularly vulnerable due to their low reproductive rate and ease of capture, resulting in severe overexploitation across their range (Lim and Ng 2007). Consequently, the IUCN outlined a population decline exceeding 50% over the next three generations, underscoring the urgent need for evidence‐based conservation interventions to curb illegal trade and habitat loss (Mahmood et al. 2019).

The Indian pangolin ( *Manis crassicaudata* ) is subject to massive pressure from illegal hunting, driven by demand for its meat and keratin scales at both local and international levels (Mahmood et al. 2012; Challender 2011; Challender et al. 2015; Heinrich et al. 2016; Baillie et al. 2015). Historically hunted for subsistence, particularly in parts of South and East Asia, contemporary exploitation is increasingly linked to organized wildlife trade networks supplying traditional medicine markets, where scales are used in whole or powdered form (CITES 2000).

In Pakistan, assessments of population status remain regulated by limited baseline data, weak enforcement capacity, and the species' cryptic, solitary, and nocturnal behavior, which collectively hinder direct detection and monitoring (Irshad et al. 2015; Akrim et al. 2017). Declining encounter rates and reduced field sightings suggest ongoing population decline, yet the absence of systematic distributional and habitat‐use data, particularly in northern Khyber Pakhtunkhwa, limits the ability to evaluate trends or identify priority conservation areas (Gaudin et al. 2006). Improving knowledge of spatial distribution and ecological correlates of occurrence is therefore critical for informing effective conservation planning and mitigating further losses.

This study investigates the spatial distribution, burrow‐based abundance patterns, habitat associations, and anthropogenic threats affecting the Indian pangolin ( *Manis crassicaudata* ) in District Buner, within Pakistan's Lesser Himalayan region. By addressing critical gaps in baseline ecological information, the findings provide an evidence base to support targeted, context‐specific conservation and management strategies.

## 2 Material and Methods

### 2.1 Study Area

The Buner district, situated in Khyber Pakhtunkhwa, Pakistan, encompasses an area of approximately 1865 km^2^. It is positioned between the latitudinal coordinates of 34.43° N and 34.71° N and the longitudinal coordinates of 72.45° E and 72.75° E. The district's elevation varies from 366 to 2911 m above sea level. The landscape of Buner is characterized by a blend of flat plains, rolling hills, and steep, mountainous regions. The lower areas are suitable for agriculture, while the higher altitudes are marked by rocky formations. Buner serves as an important corridor connecting the Peshawar Valley, Malakand, Lower Swat, Hazara Division, and Upper Indus Valley. Buner is divided into six tehsils: Daggar, Gagra, Mandanr, Khudu Khel, Gadezai, and Chagarzai. Most people live in rural areas and rely on subsistence farming to survive. The main crops are wheat, corn, barley, rice, sugarcane, and seasonal vegetables. The region has a bimodal climate characterized by cold winters (temperatures drop to −3°C in December and January) and hot summers (peaking between 40°C and 44°C from May to July) (Naveed et al. 2014). The annual rainfall ranges from 672 to 1198 mm, supporting both natural vegetation and rain‐fed farming. Hydrologically, the district is drained by seasonal streams that influence the local vegetation patterns (Khan et al. 2012). The flora comprises a diverse mix of subtropical and temperate species, including *Acacia modesta*, *Berberis lycium*, *Dodonaea viscosa* , *Olea ferruginea* , *Pinus roxburghii*, *Morus* spp., and *Eucalyptus* spp., among others. The common herbaceous species is *Adhatoda vasica* . Faunal diversity encompasses both bird and mammal species, including black and gray partridges, Chukar, Koklass, and Kalij pheasants, as well as gray goral, porcupine, jackal, red fox, Indian pangolin, Himalayan palm civet, wild boar, and rhesus monkeys (Naveed et al. 2014).

### 2.2 Methodology

The study was conducted from October 2022 to September 2023 in District Buner, Khyber Pakhtunkhwa, covering approximately 1865 km^2^ and including six tehsils (subdistricts). To ensure thorough spatial coverage, the entire district was overlaid with 2 × 2 km grid cells, totalling approximately 460. Using a systematic random sampling method, every third grid was selected, resulting in approximately 160 samples. The area was divided into three elevation zones: low (366–914 m), moderate (915–1676 m), and high (1677–2286 m), with grids proportionally allocated across these elevation levels and tehsils to account for ecological diversity and site access. Eighteen sampling sites were deliberately chosen based on habitat suitability, with three sites per tehsil. This selection was validated by the District Wildlife Department and local community members. To ensure methodological consistency and minimize observer bias, a trained team of five field researchers carried out the surveys (Perera et al. 2022).

At each study site, researchers conducted systematic surveys along three transects, each approximately three kilometers long and between 30 and 50 m wide, depending on the terrain. These surveys took place in the early morning and late evening to coincide with the crepuscular activity of Indian pangolins. The aim was to collect both direct and indirect evidence of the species' presence. Direct evidence came from live sightings, while indirect signs included burrows, feeding marks, tracks, and scats. At the same time, habitat assessments were conducted using circular plots with a 100‐m radius (~0.0314 km^2^), randomly placed within each grid cell (Waseem et al. 2020). These assessments recorded various environmental variables, such as elevation, slope, aspect, terrain type, vegetation, canopy cover, undergrowth density, and distances to water sources, roads, and human settlements. This combined approach enables the systematic detection of species and the quantitative analysis of environmental factors across different landscape gradients (Karawita et al. 2018).

The purpose of collecting field data was to identify both direct and indirect signs of Indian pangolins ( *Manis crassicaudata* ). These signs included feeding and resting burrows, abandoned burrows, footprints, claw marks, scats, and occasional visual sightings. Active burrows showed fresh signs such as clear footprints, claw marks, feces, loose soil, and remnants of termites or ants at the entrance. Inactive burrows lacked these indicators and were often covered with spider webs, grass, or leaf litter. Feeding burrows were smaller and shallower, with fresh ones showing recently dug soil and circular holes, while older ones looked weathered and undisturbed. Resting burrows were deeper and had wider entrances. The number of active resting burrows observed along the transects was used to estimate local population size (Mahmood et al. 2019).

The environmental variables at each site were systematically recorded using standardized equipment. Elevation and geographic coordinates were captured with a Garmin eTrex 10 GPS device. The slope was measured in degrees with a clinometer, and the aspect was determined with a compass, then categorized as either warmer (south‐facing) or cooler (north‐facing). Canopy cover was estimated using both a spherical densitometer and the Gap Light Analysis Mobile Application (GLAMA). The undergrowth density was visually assessed and classified into three categories: low (0%–25%), medium (26%–60%), and high (61%–100%). Proximity to water sources, roads, and human settlements was measured with GPS and laser rangefinders. The presence of rock boulders, which could serve as potential burrow shelters, was noted through visual inspection. These environmental characteristics were analyzed to assess habitat associations and ecological preferences of the species (Karawita et al. 2018).

Burrow data were used to infer relative abundance patterns and spatial variation in Indian pangolin ( *Manis crassicaudata* ) activity across stratified sampling sites, rather than to estimate absolute population size. Only active resting burrows exhibiting fresh signs, such as footprints, claw marks, feces, loose soil, and foraging remains, were recorded to minimize false positives. Burrows were treated as detection units reflecting pangolin activity and site use, acknowledging that individual pangolins may use multiple burrows over time (Mohapatra and Panda 2014). Burrow characteristics, including depth, entrance dimensions, orientation, and surrounding vegetation, were documented to ensure consistent identification and classification.

To assess anthropogenic threats and community perceptions, a structured questionnaire survey was conducted across six tehsils of District Buner. Eighteen villages (three per tehsil) were selected based on habitat suitability and confirmed or suspected pangolin presence. Fifteen households were randomly sampled in each village, yielding 270 respondents, with a minimum of 250 valid responses retained for analysis. Participants included hunters, shepherds, farmers, shopkeepers, and students, and Supporting Information on poaching incidents was obtained from Wildlife Department records.

The survey collected qualitative and quantitative data on demographic characteristics (age, gender, education, occupation, Indigenous/nonindigenous status), perceived pangolin population trends, conservation attitudes, and locally observed threats, following Newing (2011). Species recognition was facilitated using photographic guides adapted from human–wildlife conflict studies. Interviews were conducted in Pashto and Urdu. Attitudes toward pangolin conservation were categorized as harmful, positive, or neutral/unsure following Kansky et al. (2014). Spatial data were processed using ArcGIS Pro to visualize burrow distribution and threat patterns, and independent *t*‐tests were used to evaluate differences in environmental variables between burrow types and elevation categories.

## 3 Results

### 3.1 Spatial Distribution

The Indian pangolin ( *Manis crassicaudata* ) presence was recorded exclusively within low‐ to mid‐elevation zones of District Buner. Confirmed records ranged from 387 m at Totali (Tehsil Khudkheil) to 1291 m at Kandar Katai (Tehsil Mandanr). Indirect signs, including feeding, inactive, and resting burrows, were most frequently documented within the moderate elevation zone (915–1300 m), followed by the low elevation zone (366–914 m). No burrows, tracks, feeding signs, or direct sightings were recorded above 1300 m elevation.

**FIGURE 1:**
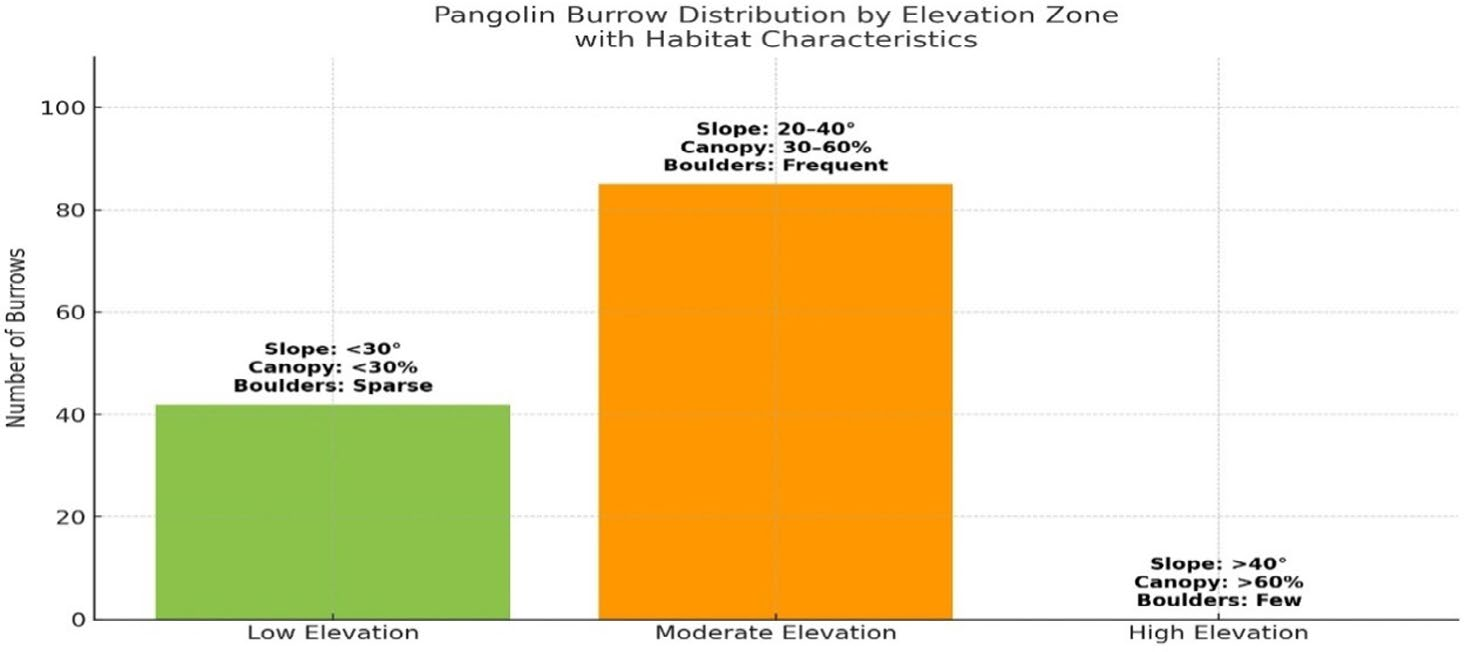
Distribution of burrows across elevation zones, with associated habitat features.

High‐elevation areas (> 1300 m), particularly within the tehsils of Gadezai and Chagharzai, showed a complete absence of pangolin presence indicators. These areas were characterized by dense stands of *Pinus roxburghii*, *Juglans regia* , and *Quercus incana* , steeper slopes, and cooler microclimatic conditions. In total, 127 burrows were documented across surveyed grids, indicating a clear restriction of pangolin activity to specific elevation bands and associated habitat types (Figure 1).

### 3.2 Direct Sightings

**FIGURE 2:**
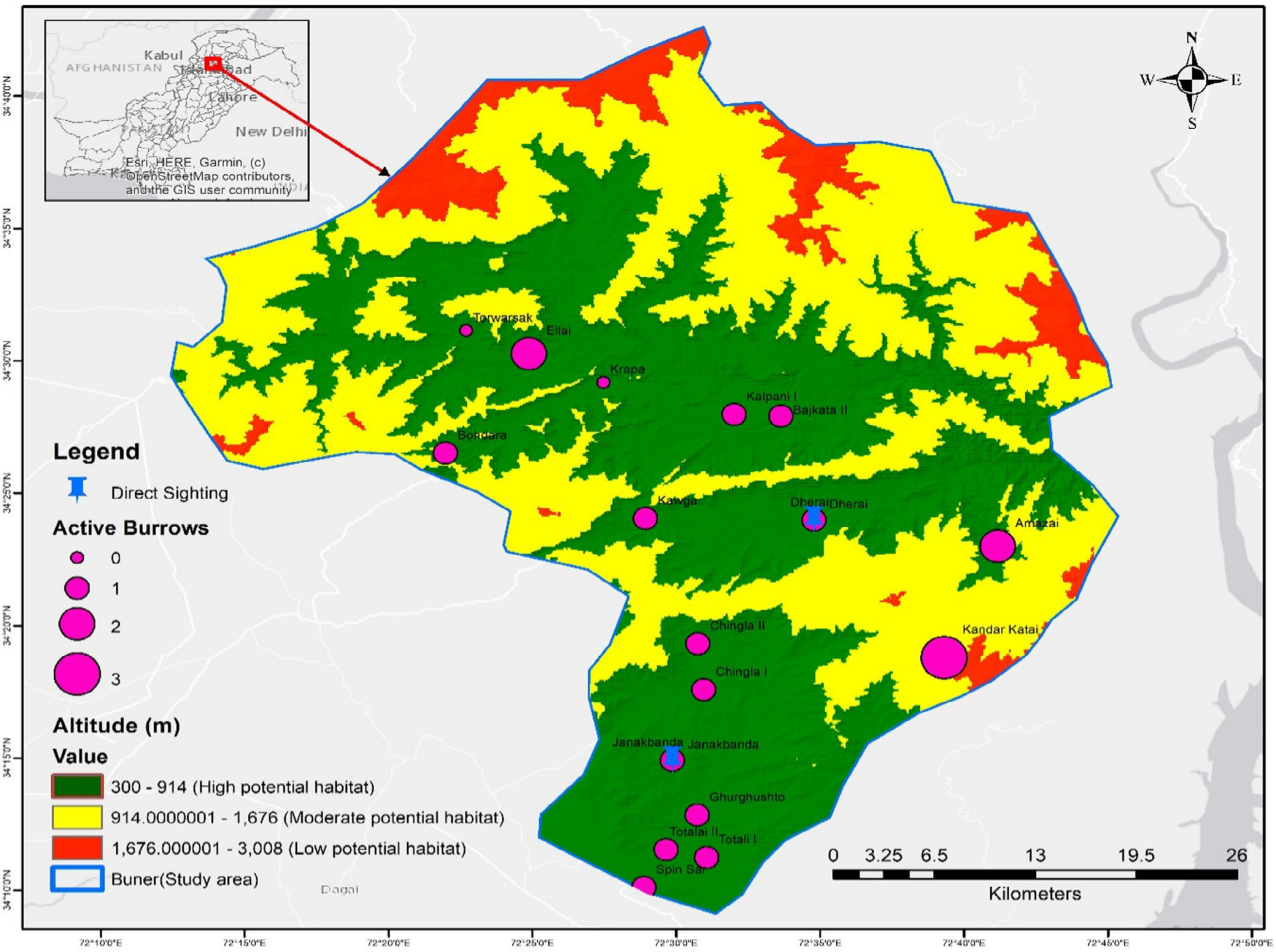
Spatial distribution of potential sites, direct sightings and active burrows in the study area.

The two direct sightings of Indian pangolins were recorded during the study period. The first sighting occurred at 19:45 during winter in Janakbanda village (Ghurghushto Union Council), Tehsil Khudkheil, at an elevation of 387 m. The individual was observed near a graveyard during nocturnal activity before retreating into the surrounding vegetation. Residents confirmed repeated sightings at this location. The second sighting was recorded at 20:41 during the summer in Dherai village (Norezai Union Council), Tehsil Gagra, at approximately 730 m elevation. The individual was observed near agricultural fields and was briefly captured by residents before being released by the Wildlife Department. These sightings provided independent confirmation of species presence in areas where active burrows were also documented (Figure 2).

### 3.3 Spatial Trends Across Tehsils and Sites

The pangolin presence varied markedly among the six tehsils and was closely associated with elevation, habitat structure, and terrain characteristics. Tehsil Mandanr recorded the highest diversity of burrow types, including feeding, inactive, and resting burrows, primarily within scrub‐dominated habitats containing rocky substrates. Tehsil Gagra also exhibited consistent pangolin activity, including feeding burrows and direct sightings, particularly in moderately elevated areas with relatively low disturbance. In Tehsil Khudkheil, burrows were mainly concentrated in cultivated landscapes and open scrub habitats at lower elevations. Tehsil Daggar showed limited evidence of pangolin activity, with a small number of feeding and inactive burrows recorded primarily along habitat margins. No evidence of pangolin presence was detected in the high‐elevation tehsils of Gadezai and Chagharzai. Spatial variation in burrow occurrence across tehsils is summarized as shown in Table 1.

**TABLE 1:** Tehsil‐wise distribution of Indian pangolin burrows in District Buner.

Tehsil	Elevation zone	No. of sites	Feeding burrows (*n*)	Inactive burrows (*n*)	Resting burrows (*n*)	Total burrows (*n*)	Burrow‐based activity category
Khudkheil	Low	3	17	8	2	27	Present
Mandanr	Moderate	3	26	9	5	40	High activity
Gagra	Moderate	3	24	10	4	38	High activity
Daggar	Moderate	3	14	6	2	22	Low activity
Gadezai	High	3	0	0	0	0	Absent
Chagharzai	High	3	0	0	0	0	Absent
Total	—	18	81	33	13	127	—

### 3.4 Distribution of Pangolin Burrows Across Tehsil

A total of 127 burrows were recorded across four tehsils and classified as feeding burrows (*n* = 81; 63.8%), inactive or abandoned burrows (*n* = 33; 26.0%), and active resting (living) burrows (*n* = 13; 10.2%), based on diagnostic field indicators such as freshness, claw marks, soil disturbance, and evidence of recent use.

In the Tehsil Mandanr, the highest number of total burrows (*n* = 40), including the greatest number of feeding (*n* = 26) and resting burrows (*n* = 5). Tehsil Gagra followed with 38 burrows, predominantly feeding types (*n* = 24). Tehsil Khudkheil recorded 27 burrows, mainly feeding burrows (*n* = 17), while Tehsil Daggar recorded 22 burrows with limited evidence of recent activity. No burrows were recorded in Gadezai or Chagharzai, as in (Table 2).

**TABLE 2:** Distribution and measurements of active pangolin burrows in District Buner.

Tehsil	Active resting burrows (*n*)	Entrance width (in.)	Entrance height (in.)	Burrow depth (in.)
Khudkheil	2	9.32 ± 0.04	9.01 ± 0.06	47.30 ± 2.65
Mandanr	5	9.40 ± 0.06	9.08 ± 0.08	49.10 ± 2.90
Gagra	4	9.38 ± 0.07	9.05 ± 0.07	49.85 ± 3.10
Daggar	2	9.35 ± 0.05	9.02 ± 0.05	48.25 ± 2.60
Overall	13	9.36 ± 0.05	9.04 ± 0.07	48.62 ± 2.97

The feeding burrows had a mean entrance width of 9.10 ± 0.14 in., a mean height of 9.02 ± 0.18 in., and a mean depth of 14.72 ± 1.25 in. Entrance shapes were predominantly circular or elliptical. Burrow depth varied among tehsils, with deeper burrows recorded in Mandanr and Gagra and comparatively shallower burrows in Daggar.

**FIGURE 3:**
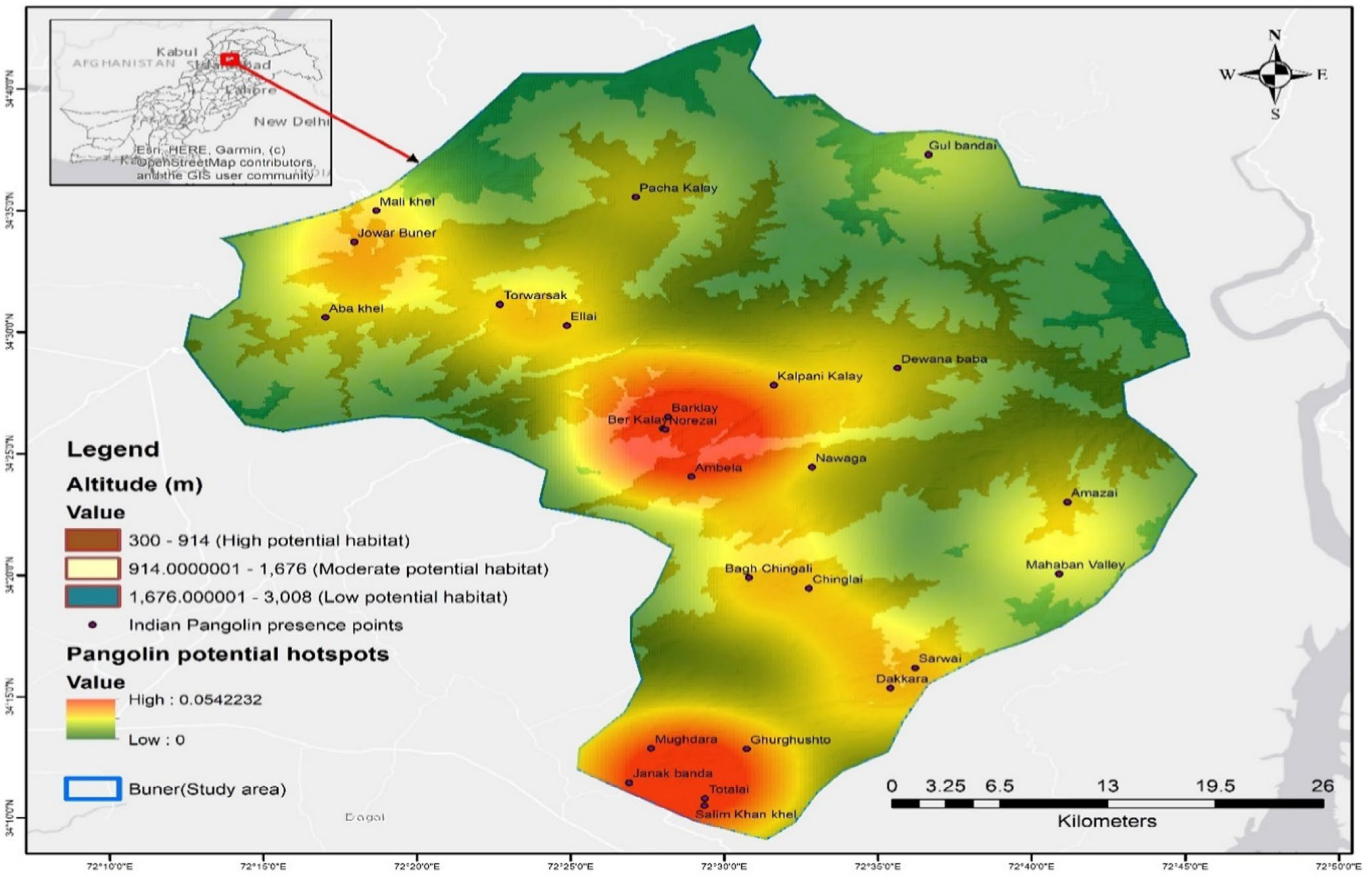
Spatial distribution of potential hotspots of pangolins in the study area.

The active resting burrows (*n* = 13) were characterized by greater depth, fresh claw marks, and pronounced soil disturbance. These burrows had a mean entrance width of 9.36 ± 0.05 in., a mean height of 9.04 ± 0.07 in., and a mean depth of 48.62 ± 2.97 in. (Table 3). Resting burrows were most frequently recorded in Mandanr (*n* = 5) and Gagra (*n* = 4), with fewer occurrences in Khudkheil and Daggar (*n* = 2 each) and none in the high‐elevation tehsils (Figure 3).

**TABLE 3:** Comparison of environmental and structural characteristics between resting and feeding burrows.

Variable	Resting burrows (*n* = 13)	Feeding burrows (*n* = 81)	Test statistic, *t*	*p*
Elevation (m)	1129.18	954.68	7.44	< 0.0001***
Slope (°)	22.01	20.19	1.21	0.241
Canopy cover (%)	56.18	50.95	1.66	0.115
Undergrowth (%)	53.16	47.03	2.15	0.040*
Distance to water (m)	149.77	174.56	−3.03	0.006**
Distance settlement (m)	313.16	274.11	2.33	0.033*
Entrance width (cm)	24.76	20.08	4.09	0.0004***
Entrance height (cm)	18.95	15.80	4.44	0.0001***
Burrow depth (cm)	93.62	77.08	3.49	0.003**

* *p* < 0.05.

** *p* < 0.01.

*** *p* < 0.001.

Burrow occurrence showed consistent associations with specific habitat features. Most burrows were recorded in areas with moderate canopy cover (41%–70%) and dense undergrowth (51%–100%). Burrows were predominantly located on gentle to moderate slopes (< 30°), and 64% of resting burrows were situated near rock boulders. Distance analysis indicated that most burrows occurred within 200–500 m of roads, human settlements, and water sources. Aspect analysis revealed a strong preference for cooler north‐ and east‐facing slopes, where all resting burrows and the majority of feeding (*n* = 74) and inactive burrows (*n* = 33) were recorded. In contrast, only a small number of feeding burrows (*n* = 7) were located on warmer south‐facing slopes (Table 3).

### 3.5 Phyto‐Association and Photo‐Habitat Analysis

The Pangolin burrows exhibited clear associations with specific vegetation types. In Mandanr and Gagra, resting burrows were frequently located beneath *Lantana camara* and *Punica granatum* . In Khudkheil, feeding burrows were commonly associated with *Dalbergia sissoo* and *Acacia modesta*, vegetation types known to support dense ant and termite populations.

Additional plant species recorded near burrow sites included *Zizyphus nummularia*, *Pistacia integerrima*, *Melia azedarach* , *Ficus carica* , *Eucalyptus camaldulensis* , *Morus alba* , and *Grewia optiva*. Shrub and herbaceous layers were dominated by *Carissa opaca*, *Dodonaea viscosa* , *Adhatoda vasica* , *Cynodon dactylon* , *Saccharum bengalense* , and *Cannabis sativa* . Burrow occurrence was highest in ecotonal zones between the scrub forest and subtropical pine forest. No burrows or presence indicators were recorded in upland vegetation zones dominated by *Pinus roxburghii*, *Quercus incana* , *Juglans regia* , and *Rhododendron arboreum*.

### 3.6 Threats and Community Perceptions

The community surveys revealed significant variation in conservation attitudes across demographic groups. Support for pangolin conservation was higher among educated respondents (*p* < 0.01) and Indigenous participants (55%) compared to nonindigenous respondents (33%) (*p* < 0.05). Illegal hunting, reported by 60%–70% of respondents, and habitat loss were identified as the most prevalent threats.

The Indigenous respondents more frequently cited agricultural expansion, marble mining, fires, and road construction as key drivers of habitat degradation. Several respondents reported past confiscations of pangolins by wildlife authorities, indicating ongoing enforcement activity alongside persistent illegal exploitation. Weak law enforcement capacity, low awareness, and limited community involvement were consistently identified as barriers to effective conservation.

## 4 Discussion

The Indian pangolin ( *Manis crassicaudata* ) is classified as Endangered on the IUCN Red List and is protected under CITES Appendix S1 and national wildlife legislation in Pakistan. Despite these legal safeguards, the species continues to experience intense pressure from illegal wildlife trade, particularly in Khyber Pakhtunkhwa, where demand for pangolin scales persists (Mahmood et al. 2012; Roberts 1997). Given its low reproductive rate, cryptic behavior, and ecological specialization, even localized disturbances can result in rapid declines. In northern Pakistan, including the Lesser Himalayan region, empirical data on pangolin ecology remain limited, constraining evidence‐based conservation planning. By integrating field‐based ecological data with community perceptions, this study addresses this gap through a detailed assessment of spatial distribution, burrow characteristics, habitat associations, and threats in District Buner.

### 4.1 Spatial Distribution and Habitat Use

This study provides the first spatially explicit assessment of *M. crassicaudata* distribution in District Buner and demonstrates that pangolin presence is restricted to low‐ and mid‐elevation landscapes where specific habitat conditions prevail. Burrows and other presence indicators were concentrated in moderate elevations within scrub‐dominated and mixed vegetation systems, particularly in Mandanr and Gagra tehsils. These areas are characterized by heterogeneous terrain, loose soils, moderate canopy cover, and the presence of boulders—features that collectively facilitate burrow construction and access to prey.

In contrast, the absence of pangolin signs in the high‐elevation tehsils of Gadezai and Chagharzai reflects a clear spatial limitation within the surveyed landscape. These areas are dominated by steep slopes, dense forest cover, and cooler microclimates, conditions that are less suitable for burrowing and foraging by a myrmecophagous mammal. Comparable elevational patterns have been reported from Nepal and northern India, where pangolins are largely confined to landscapes with intermediate canopy openness and relatively stable thermal environments (Karawita et al. 2018; Perera 2017). Importantly, these findings are interpreted as site‐specific patterns based on survey coverage and do not represent generalized elevational limits for the species.

Burrow‐type differentiation further clarifies habitat use. Feeding burrows were shallow and widely distributed, often occurring closer to water sources and settlements, whereas resting burrows were significantly deeper and located in areas with denser undergrowth and greater separation from anthropogenic activity. Statistically significant associations with elevation, undergrowth cover, and distance from settlements and water sources indicate selective placement of resting burrows, likely reflecting requirements for security and microclimatic stability (Perera 2017). These patterns are consistent with earlier studies in Pakistan that document functional differentiation between foraging and refuge sites in pangolins (Mahmood et al. 2015, 2019).

### 4.2 Direct Sightings and Species Confirmation

The indirect evidence formed the primary basis for detecting pangolins in this study, reflecting the species' cryptic and nocturnal behavior. The two confirmed direct sightings provided independent validation of burrow‐based inference. Both sightings occurred at low to moderate elevations and during nocturnal hours, consistent with observations from Pakistan and other parts of South Asia (Akrim et al. 2017; Mahmood et al. 2021).

The seasonal context further supports known behavioral flexibility. Winter activity near graveyards and summer observations in agricultural landscapes suggest opportunistic use of human‐modified environments where prey resources remain available. The involvement of the Wildlife Department in responding to one encounter highlights the importance of rapid institutional intervention in mitigating harm during incidental human–pangolin interactions. Although limited in number, these sightings validate patterns inferred from burrow data and emphasize the value of community reporting in pangolin monitoring (Yasmeen et al. 2021).

### 4.3 Phyto‐Association and Habitat Structure

The vegetation composition around burrow sites revealed strong phytogeographic associations relevant to habitat suitability. Resting burrows in Mandanr and Gagra were frequently located beneath *Lantana camara* and *Punica granatum* , which provide dense ground cover and buffered microclimatic conditions. Feeding burrows in Khudkheil were commonly associated with *Dalbergia sissoo* and *Acacia modesta*, tree species known to support abundant ant and termite populations.

Burrow‐associated vegetation formed multilayered assemblages of trees, shrubs, and herbaceous cover. Ecotonal zones between scrubland and subtropical pine forest supported the highest levels of burrow activity, likely due to structural heterogeneity and prey diversity. Similar associations have been reported from Sri Lanka and central India, where pangolins preferentially occupy seminatural agro‐forest mosaics rather than closed‐canopy forests or heavily degraded landscapes (Perera 2017; Karawita et al. 2018; Mahmood et al. 2021). These findings reinforce the role of vegetation structure and prey availability in shaping pangolin habitat use.

### 4.4 Burrow‐Based Indicators of Population Status

The distribution of burrow types provides indirect insight into population status within the surveyed landscape. The predominance of feeding burrows relative to active resting burrows suggests limited availability of secure refuges or reduced breeding activity, patterns that have been interpreted elsewhere as indicators of population stress (Mahmood et al. 2015; Irshad et al. 2015). The restriction of pangolin presence to four of six tehsils further indicates spatial contraction within District Buner. Comparable declines have been documented in other regions of Pakistan, including the Pothohar Plateau and Punjab, where encounter rates and burrow densities have declined over time (Irshad et al. 2015; Mahmood et al. 2021). Given the species' slow reproductive rate and sensitivity to disturbance, sustained recovery under ongoing exploitation is unlikely without targeted conservation intervention (Challender and MacMillan 2014; Heinrich et al. 2016). These interpretations are framed explicitly as relative indicators derived from burrow‐based inference, consistent with the analytical scope of the study.

Burrow‐based inference has been widely applied in previous pangolin studies in Pakistan to assess relative abundance and spatial patterns. For example, studies from Margalla Hills National Park (Mahmood et al. 2015) and Pir Lasura National Park, Azad Jammu and Kashmir (Akrim et al. 2017) used counts of active resting burrows as indicators of pangolin abundance and distribution across elevation gradients. While such approaches do not account for detection probability or potential burrow reuse, they provide a practical and comparable framework for evaluating population status in cryptic, low‐density species. In this context, the present study adopts a conservative interpretation of burrow data as indicators of activity and habitat use rather than absolute population size, ensuring consistency with both methodological limitations and regional precedent.

### 4.5 Socioeconomic Drivers, Mining Pressure, and Community Perceptions

The community surveys indicate that anthropogenic pressures play a central role in shaping pangolin distribution and risk. Pangolins frequently forage near settlements, increasing exposure to threats such as dog attacks, road mortality, and opportunistic poaching. Misidentification as agricultural pests and beliefs surrounding the medicinal or symbolic value of pangolin scales further aggravate exploitation (Kansky et al. 2014).

Marble mining and associated infrastructure development represent a growing threat in District Buner. Quarrying activities lead to habitat loss, soil compaction, vegetation removal, and increased access to previously undisturbed areas, effects that disproportionately impact the moderate‐elevation zones identified as core pangolin habitat. Similar mining‐related pressures have been linked to a decline in wildlife turnout in other parts of Pakistan (Mahmood et al. 2021). Weak law enforcement, limited awareness, and lack of alternative livelihoods collectively reinforce exploitation despite legal protection under CITES Appendix S1 (Challender et al. 2015).

### 4.6 Implications for Conservation and Management

The findings underscore the need for an integrated, landscape‐scale conservation approach in District Buner. Moderate‐elevation scrub–pine ecotones should be prioritized for protection and restoration, as they support the highest levels of pangolin activity. Habitat restoration using native species such as *Dalbergia sissoo* , *Carissa opaca*, and *Zizyphus nummularia* may enhance prey availability and refuge quality.

Community engagement is essential for reducing illegal exploitation. Participatory monitoring, conservation education, and incentive‐based livelihood alternatives can minimize dependence on extractive activities while fostering local stewardship. Improving surveillance and enforcement in identified hotspots, particularly near mining zones and transport corridors, is critical for halting illegal trade networks. Long‐term monitoring using camera traps, environmental DNA, and spatial modeling should be institutionalized to track trends and inform adaptive management. Integrating pangolin conservation into provincial biodiversity strategies and developing a species‐specific action plan would align local efforts with Pakistan's commitments under the Convention on Biological Diversity and the Post‐2020 Global Biodiversity Framework.

## 5 Conclusion

This study presents the first comprehensive ecological and spatial assessment of the Indian pangolin in District Buner, Pakistan, demonstrating a restricted and fragmented distribution closely associated with habitat structure, vegetation composition, and human disturbance. Pangolin activity was confined to low‐ and mid‐elevation landscapes characterized by scrub‐dominated ecotones, suitable soils, and prey availability. Differences in burrow types and habitat associations highlight the species' sensitivity to environmental change and anthropogenic pressure.

Poaching, habitat degradation, and expanding marble mining activities pose substantial threats, compounded by weak enforcement and limited community awareness. An integrated conservation strategy emphasizing habitat protection, community engagement, targeted enforcement, and long‐term monitoring is essential to prevent further decline. Future research should prioritize spatial modeling and genetic connectivity analyses to support adaptive conservation strategies in a rapidly changing landscape.

## Author Contributions

**Shahrul Anuar Mohd Sah:** project administration (equal), supervision (lead), validation (lead), visualization (equal). **Muhammad Saad:** conceptualization (lead), formal analysis (lead), investigation (lead), methodology (lead), writing – original draft (equal), writing – review and editing (equal).

## Funding

The authors have nothing to report.

## Ethics Statement

Verbal consent was obtained, and no personal data were collected. Field activities were conducted according to established ethical standards and in coordination with local authorities.

## Conflicts of Interest

The authors declare no conflicts of interest.

## Supporting information


**Appendix S1:** ece373344‐sup‐0001‐AppendixS1.docx.

**Plate 1.** Old resting burrow of Indian pangolin (*Manis crassicaudata*) in the study area.
**Plate 2**. Interview with local eyewitness documenting historical presence of Indian pangolin in the study area.
**Plate 3**. Interviews with hunters and local community members regarding pangolin occurrence and trade.
**Plate 4**. Interviews with shopkeepers and local community members on trade‐related information.
**Plate 5**. Field survey with local community members for locating pangolin burrows in the study area.
**Plate 6**. Resting burrow of the Indian pangolin (*Manis crassicaudata*) located beneath a rocky outcrop.
**Plate 7**. Potential habitat of Indian pangolin (*Manis crassicaudata*) in the study area.

## Data Availability

All data supporting this study are provided as a Supporting Information for editorial and peer review. Upon acceptance, the dataset will be deposited in a public repository in accordance with ESA's Open Research Policy.
