# Supplementary material for: Spatial Distribution, Abundance, and Threats to the Indian Pangolin ( Manis crassicaudata ) in Buner District, Lesser Himalayas
Source: Ecol Evol. 2026 May 4;16(5):e73344. doi: 10.1002/ece3.73344 (PMC13139718; doi:10.1002/ece3.73344)
Supplement: Supplementary file 1 — Appendix S1: ece373344‐sup‐0001‐AppendixS1.docx. [file ECE3-16-e73344-s001.docx]

|  | **I** | **Appendices II** | **III** |
| --- | --- | --- | --- |
| RHINOCEROTIDAE Rhinoceroses |  | | |
|  | **RHINOCEROTIDAE spp.** (Except the subspecies included in Appendix II) | ***Ceratotherium simum simum*** (Only the populations of EswatiniA8, NamibiaA9 and South AfricaA8; all other populations are included in Appendix I) |  |
| TAPIRIDAE Tapirs |  | | |
|  | **TAPIRIDAE spp.** (Except the species included in Appendix II) | ***Tapirus terrestris*** |  |
| PHOLIDOTA |  | | |
| MANIDAE Pangolins |  | | |
|  | ***Manis crassicaudata Manis culionensis Manis gigantea Manis javanica Manis pentadactyla Manis temminckii Manis tetradactyla*** 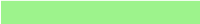 ***Manis tricuspis*** | ***Manis* spp.** (Except the species included in Appendix I) 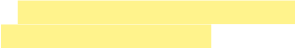 |  |
| PILOSA |  | | |
| BRADYPODIDAE Three-toed sloths |  | | |
|  |  | ***Bradypus pygmaeus***  ***Bradypus variegatus*** |  |

^A8^ The populations of Eswatini and South Africa of *Ceratotherium simum simum* are included in Appendix II for the exclusive purpose of allowing international trade in live animals to appropriate and acceptable destinations and hunting trophies. All other specimens shall be deemed to be specimens of species included in Appendix I and the trade in them shall be regulated accordingly.

^A9^ The population of Namibia of *Ceratotherium simum simum* is included in Appendix II for the exclusive purpose of allowing international trade in live animals for *in-situ* conservation only, and only within the natural and historical range of *Ceratotherium simum* in Africa. All other specimens shall be deemed to be specimens of species included in Appendix I and the trade in them shall be regulated accordingly.

*Appendices I, II & III (07/02/2025) – p. 17*
