# Supplementary figures and images for: Spatial Distribution, Abundance, and Threats to the Indian Pangolin ( Manis crassicaudata ) in Buner District, Lesser Himalayas
Source: Ecol Evol. 2026 May 4;16(5):e73344. doi: 10.1002/ece3.73344 (PMC13139718; doi:10.1002/ece3.73344)

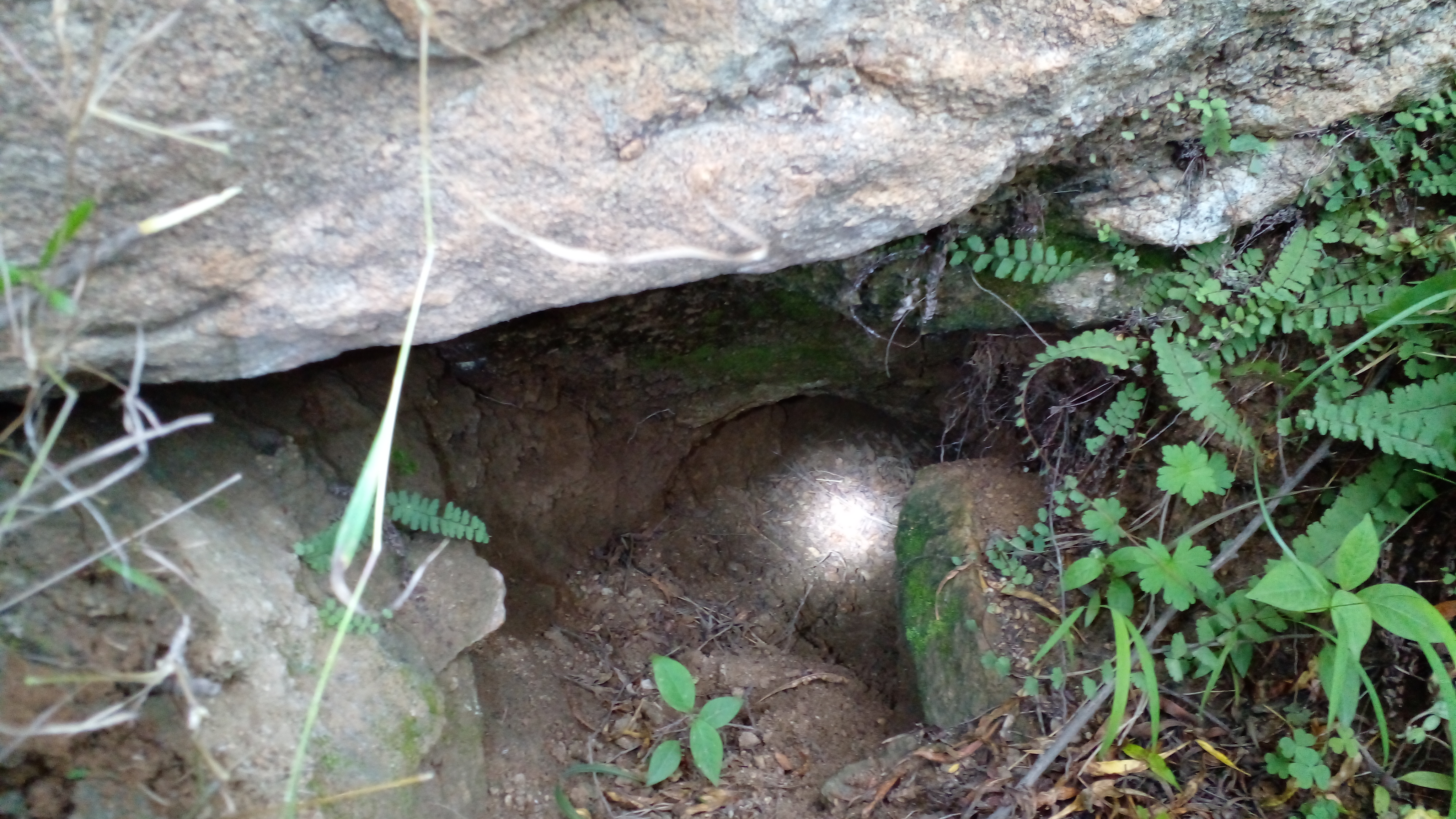

Supplement: Supplementary file 2 — Plate 1. Old resting burrow of Indian pangolin (Manis crassicaudata) in the study area. Plate 2. Interview with local eyewitness documenting historical presence of Indian pangolin in the study area. Plate 3. Interviews with hunters and local community members regarding pangolin occurrence and trade. Plate 4. Interviews with shopkeepers and local community members on trade‐related information. Plate 5. Field survey with local community members for locating pangolin burrows in the study area. Plate 6. Resting burrow of the Indian pangolin (Manis crassicaudata) located beneath a rocky outcrop. Plate 7. Potential habitat of Indian pangolin (Manis crassicaudata) in the study area. [file ECE3-16-e73344-s002.zip › Plate _pagolin 6.jpg]

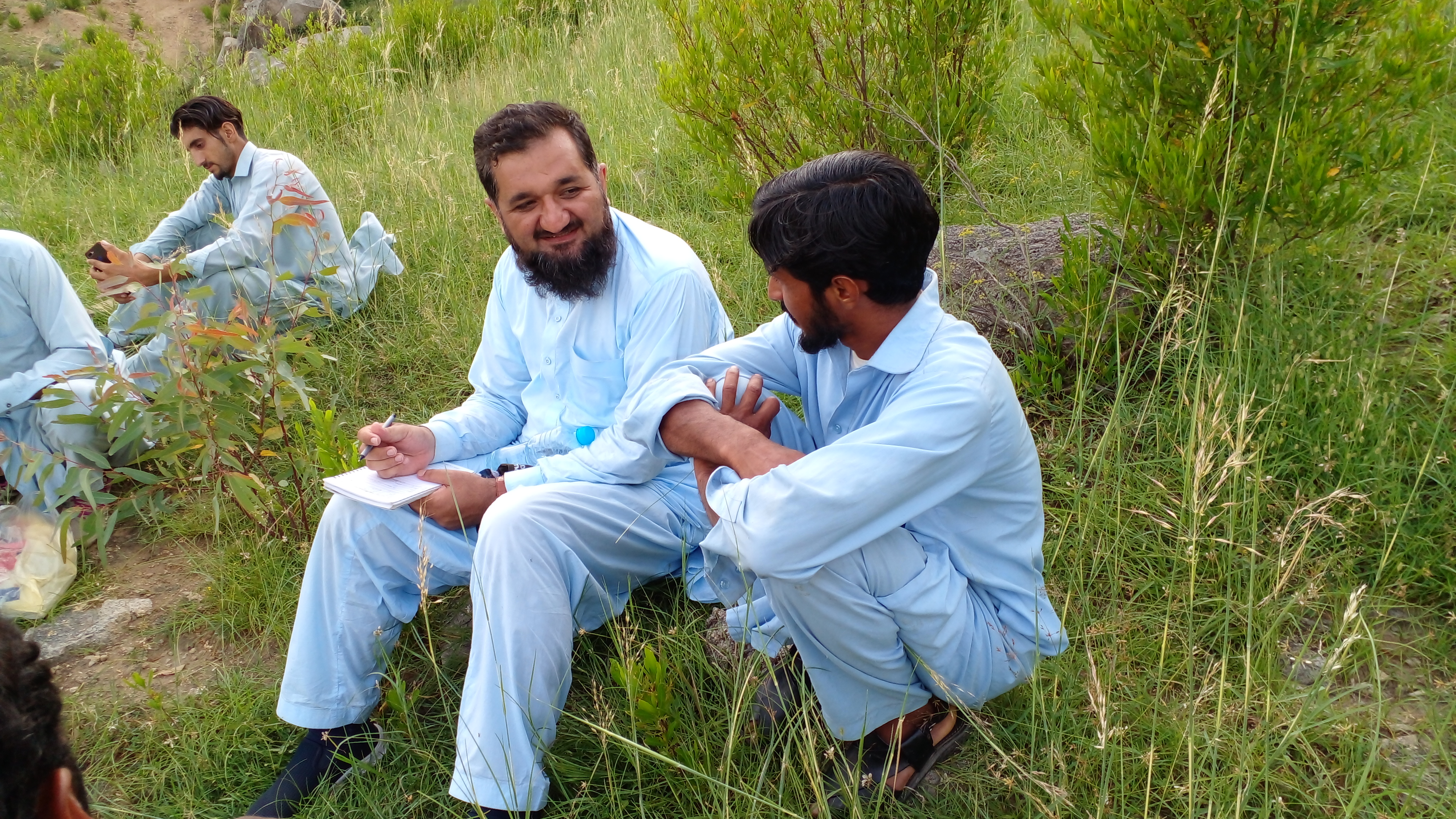

Supplement: Supplementary file 2 — Plate 1. Old resting burrow of Indian pangolin (Manis crassicaudata) in the study area. Plate 2. Interview with local eyewitness documenting historical presence of Indian pangolin in the study area. Plate 3. Interviews with hunters and local community members regarding pangolin occurrence and trade. Plate 4. Interviews with shopkeepers and local community members on trade‐related information. Plate 5. Field survey with local community members for locating pangolin burrows in the study area. Plate 6. Resting burrow of the Indian pangolin (Manis crassicaudata) located beneath a rocky outcrop. Plate 7. Potential habitat of Indian pangolin (Manis crassicaudata) in the study area. [file ECE3-16-e73344-s002.zip › Plate _pangolin 5.jpg]

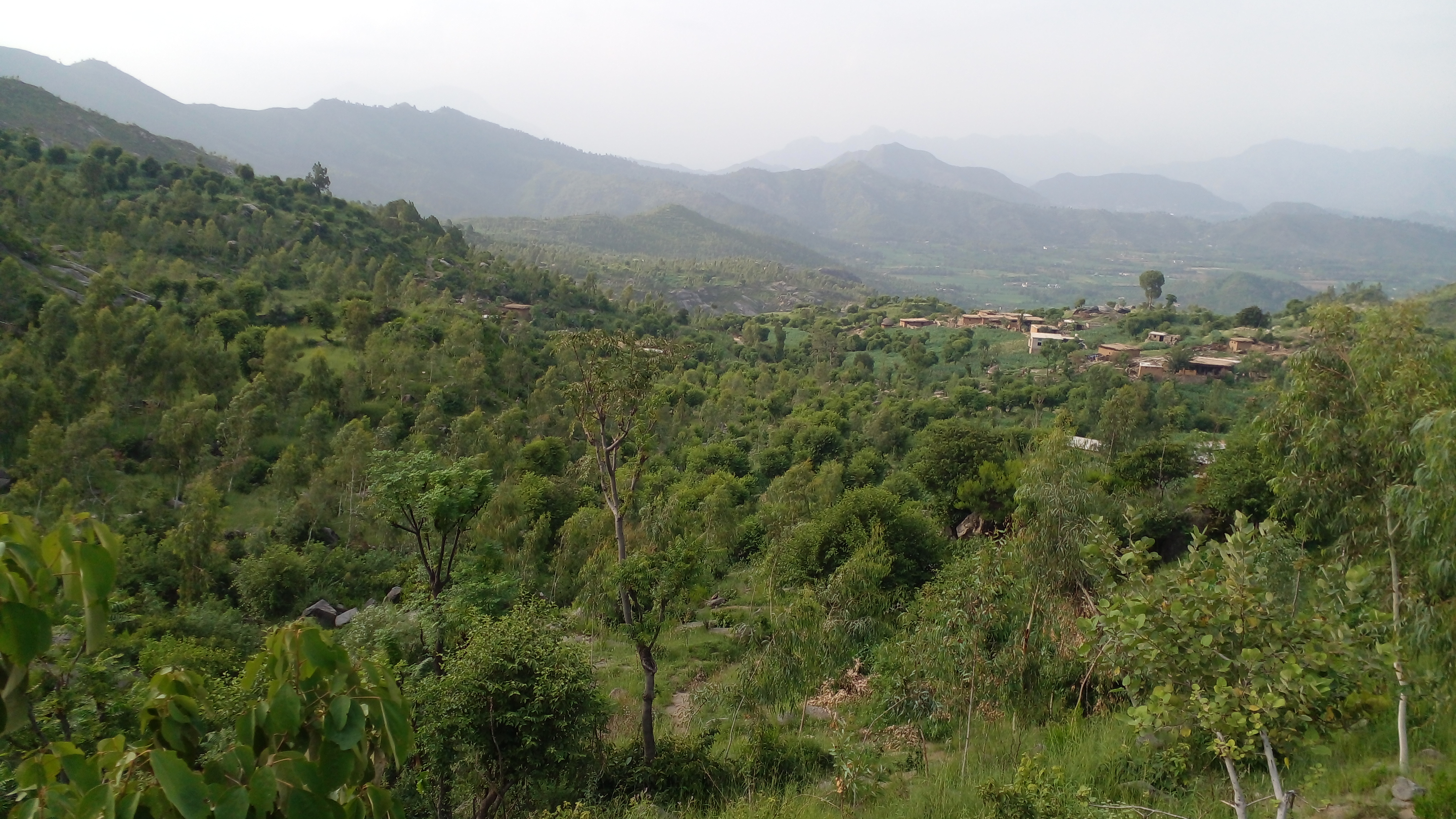

Supplement: Supplementary file 2 — Plate 1. Old resting burrow of Indian pangolin (Manis crassicaudata) in the study area. Plate 2. Interview with local eyewitness documenting historical presence of Indian pangolin in the study area. Plate 3. Interviews with hunters and local community members regarding pangolin occurrence and trade. Plate 4. Interviews with shopkeepers and local community members on trade‐related information. Plate 5. Field survey with local community members for locating pangolin burrows in the study area. Plate 6. Resting burrow of the Indian pangolin (Manis crassicaudata) located beneath a rocky outcrop. Plate 7. Potential habitat of Indian pangolin (Manis crassicaudata) in the study area. [file ECE3-16-e73344-s002.zip › Plate _pangolin 7.jpg]
